# Theta and alpha EEG frequency interplay in subjects with mild cognitive impairment: evidence from EEG, MRI, and SPECT brain modifications

**DOI:** 10.3389/fnagi.2015.00031

**Published:** 2015-03-20

**Authors:** Davide V. Moretti

**Affiliations:** Istituto di Ricovero e Cura a Carattere Scientifico San Giovanni di Dio – Fatebenefratelli, Brescia, Italy

**Keywords:** theta, alpha, EEG, SPECT, MRI, midl cognitive impairment

## Abstract

**Background:** Temporo-parietal and medial temporal cortex atrophy are associated with mild cognitive impairment (MCI) due to Alzheimer disease (AD) as well as the reduction of regional cerebral blood perfusion in hippocampus. Moreover, the increase of EEG alpha3/alpha2 power ratio has been associated with MCI due to AD and with an increase in theta frequency power in a group of subjects with impaired cerebral perfusion in hippocampus.

**Methods:** Seventy four adult subjects with MCI underwent clinical and neuropsychological evaluation, electroencephalogram (EEG) recording and high resolution 3D magnetic resonance imaging (MRI). Among the patients, a subset of 27 subjects underwent also perfusion single-photon emission computed tomography and hippocampal atrophy evaluation. Alpha3/alpha2 power ratio as well as cortical thickness was computed for each subject. Three MCI groups were detected according to increasing tertile values of alpha3/alpha2 power ratio and difference of cortical thickness among the groups estimated.

**Results:** Higher alpha3/alpha2 power ratio group had wider cortical thinning than other groups, mapped to the Supramarginal and Precuneus bilaterally. Subjects with higher alpha3/alpha2 frequency power ratio showed a constant trend to a lower perfusion than lower alpha3/alpha2 group. Moreover, this group correlates with both a bigger hippocampal atrophy and an increase of theta frequency power.

**Conclusion:** Higher EEG alpha3/alpha2 power ratio was associated with temporo-parietal cortical thinning, hippocampal atrophy and reduction of regional cerebral perfusion in medial temporal cortex. In this group an increase of theta frequency power was detected inMCI subjects. The combination of higher EEG alpha3/alpha2 power ratio, cortical thickness measure and regional cerebral perfusion reveals a complex interplay between EEG cerebral rhythms, structural and functional brain modifications.

## Introduction

The MCI commonly represent the ‘at-risk’ state of developing dementia. In neurodegenerative disorders, like AD or other dementias, the brain networks modifies many years before clinical manifestations (Dubois et al., 2007; Hampel et al., 2007; Albert et al., 2011; Galluzzi et al., 2013). Recent MRI studies have demonstrated that a large neural network is altered in subjects with prodromal AD, including precuneus, medial temporal, parietal, and frontal cortices (Frisoni et al., 2006, 2007, 2008, 2009; Van Strien et al., 2009; Frisoni, 2012). In particular, subjects with cognitive decline have shown early atrophy and loss of gray matter in cortical specific brain areas (Frisoni et al., 2007, 2009), including hippocampal, medial temporal and parietal lobes. In the conceptual frame of the integration of biomarkers for an early and highly predictive diagnosis, the EEG could be a reliable tool (Missonnier et al., 2010). Indeed, it is widely accepted that the cerebral EEG rhythms reflect the underlying brain network activity (Steriade, 2006). As a consequence, modifications in EEG rhythms could be an early sign of disease associated with AD-related structural and functional networks. In particular, the study of alpha rhythm seems to be a very suitable tool to detect relationship between structural and functional brain networks. Previous studies has convincingly demonstrated that there are thalamo-cortical and cortico-cortical components which interact in the generation of cortical alpha rhythms (Lopes da Silva et al., 1980). According to the seminal paper of Lopes Da Silva, the disrupture of long-range network, inpinguing on low alpha frequency, is replaced by an increase in higher frequency (upper alpha) synchronization, which is based on narrower cell assemblies activity. Furthermore, the dynamic behavior of alpha rhythm is apparently due to some combination of global and local processes. The global processes appear to be analogous to large-scale coherent EEG observed in low alpha frequency, whereas the local processes seem to be analogous to the smaller (mesoscopic) scale columnar dynamics, observed in upper alpha frequency (Ingber and Nunez, 2011). Given the well-known loss of brain network complexity in AD pathology (Nunez, 1989; Stam et al., 2005), it is highly conceivable an impairment of long-range connectivity pathways, replaced by short-range, downsized, cell assemblies connections, resulting in a decrease of low alpha and an increase of upper alpha frequency power. Recent single-photon emission computed tomography (SPECT) studies have demonstrated that a large neural network is altered in subjects with prodromal AD, including precuneus, medial temporal, parietal and frontal cortices (Rodriguez et al., 1999). For instance, selective regional cerebral blood perfusion (rCBF) reductions in the left hippocampus and parahippocampal gyrus and in extended areas of cerebral association cortex were demonstrated in a 2-years follow-up clinical study with rCBF-SPECT (Pupi et al., 2005). Cross-sectional studies have shown rCBF and regional metabolic rates of glucose (rCMRgl) reductions in the resting state throughout the cortex in AD, involving distinctive brain structures such as the posterior cingulate/precuneus, temporoparietal, and frontal cortices (Frisoni, 2012). A positive SPECT scan raised the likelihood of diagnosing pathological AD from 84%, as defined by clinical diagnosis, to 92% (Frisoni, 2012). Recent results show that there is a hippocampal rCBF hypoperfusion in patients with mild AD (Moretti et al., 2012b), as well as that baseline SPECT can support outcome prediction in subjects with MCI (Pupi et al., 2005). Of note, rCBF (bilateral parietal perfusion) and qEEG (especially the slowest frequencies, i.e., 2–5.5 Hz) are confirmed to be good descriptors of AD severity. It is especially noteworthy that bilateral hippocampal rCBF reduction was the perfusional index best correlated with both cognitive performance and qEEG (Rodriguez et al., 1999). Recent studies confirms the relationship of higher alpha3/alpha2 frequency power ratio with a smaller hippocampal volume and a lower cerebral perfusion (Moretti et al., 2013b). Recently, it has been demonstrated that temporo-parietal and medial temporal cortex atrophy are associated with mild cognitive impairment (MCI) due to Alzheimer disease (AD) as well as the reduction of regional cerebral perfusion in hippocampus. Moreover, the increase of EEG alpha3/alpha2 power ratio has been associated with MCI due to AD and with an increase in theta frequency power in a group of subjects with impaired cerebral perfusion in hippocampus (Moretti et al., 2009, 2011a,b, 2012a,b, 2013a,b).

In this study, we investigated the possible interactions between brain rhythms and their associations with data morphostructural in an attempt to investigate the anatomical and pathophysiological alterations at the base of the prodromal phase of AD.

## Materials and Methods

### Subjects

For the present study, 74 subjects with MCI were recruited from the memory Clinic of the Scientific Institute for Research and Care (IRCCS) of Alzheimer’s and psychiatric diseases ‘Fatebenefratelli’ in Brescia, Italy. All experimental protocols had been approved by the local ethics committee. Informed consent was obtained from all participants or their caregivers, according to the Code of Ethics of the World Medical Association (Declaration of Helsinki).

#### Diagnostic Criteria

Patients were selected from a prospective study on the natural history of cognitive impairment (the translational outpatient memory clinic—TOMC study) carried out in the outpatient facility of the National Institute for the Research and Care of Alzheimer’s Disease (IRCCS Istituto Centro San Giovanni di Dio Fatebenefratelli, Brescia, Italy). The diagnosis of prodromal AD has been made according recent guidelines (Dubois et al., 2007; Albert et al., 2011; Galluzzi et al., 2013).

The project was aimed to study the natural history of non-demented persons with apparently primary cognitive deficits, i.e., deficits not due to psychic (anxiety, depression, etc.) or physical (hypothyroidism, vitamin B12 and folate deficiency, uncontrolled heart disease, uncontrolled conditions (diabetes, etc.) in the absence of functional impairment. The selection criteria has the aim to include as much as possible primary prodromal dementia due to neurodegenerative disorders. Demographic and cognitive features of the subjects in study are summarized in **Table 1**.

**Table 1:** Demographic and cognitive characteristics in the whole sample, according to increased levels of alpha3/alpha2 Numbers denote mean ± SD, number, and [range].

	Alpha3/alpha2			
	High	Middle	Low	*p*
**Demographic and clinical futures**
Number of subjects	18	38	18	—
Age, years	70.4 ± 6.7 [60–85]	68.4 ± 8.2 [52–83]	70.4 ± 7.4 [57–80]	0.55
Sex, female	13 (%)	24 (%)	14 (%)	0.51
Education, years	6.6 ± 3.6 [4–18]	7.6 ± 3.7 [3–17]	8.3 ± 4.7 [3–18]	0.42
Mini Mental State Exam	27 ± 1.7	27.4 ± 1.3	26.9 ± 1.2	0.46
Alpha3/alpha2	1.29 ± 0.1 [1.17–1.52]	1.08 ± 0.0 [1–1.16]	0.9 ± 0.1 [0.77–0.98]	**0.000**

*p denotes significance on ANOVA. Bold value indicates statistical significant results*.

Patients were rated with a series of standardized diagnostic and severity instruments, including the Mini-Mental State Examination (MMSE; Folstein et al., 1975), the Clinical Dementia Rating Scale (CDRS; Hughes et al., 1982), the Hachinski Ischemic Scale (HIS; Rosen et al., 1980) and the Instrumental and Basic Activities of Daily Living (IADL, BADL; Lawton and Brodie, 1969). In addition, patients underwent diagnostic neuroimaging procedures (magnetic resonance imaging, MRI), and laboratory testing to rule out other causes of cognitive impairment. These inclusion and exclusion criteria for MCI were based on previous seminal studies (Petersen et al., 2001; Portet et al., 2006; Dubois et al., 2007). Inclusion criteria of the study were all of the following: (i) complaint by the patient, or report by a relative or the general practitioner, of memory or other cognitive disturbances; (ii) MMSE score of 24–27/30, or MMSE of 28 and higher plus low performance (score of 2–6 or higher) on the clock drawing test (Lezak et al., 2004); (iii) sparing of IADL, BADL or functional impairment steadily due to causes other than cognitive impairment, such as physical impairments, sensory loss, gait or balance disturbances, etc. Exclusion criteria were any one of the following: (i) patients aged 90 years and older (no minimum age to participate in the study); (ii) history of depression (from mild to moderate or major depression) or juvenile-onset psychosis; (iii) history or neurological signs of major stroke; (iv) other psychiatric diseases, overt dementia, epilepsy, drug addiction, alcohol dependence; (v) use of psychoactive drugs, including acetylcholinesterase inhibitors or other drugs enhancing brain cognitive functions or biasing EEG activity; and (vi) current or previous uncontrolled or complicated systemic diseases (including diabetes mellitus), or traumatic brain injuries. All subjects were right-handed.

All patients underwent: (i) semi-structured interview and – whenever possible – with another informant (usually, the patient’s spouse or a child of the patient) by a geriatrician or neurologist; (ii) physical and neurological examinations; (iii) performance-based tests of physical function, gait and balance; (iv) neuropsychological battery assessing memory (Babcock Story Recall – Rey–Osterrieth Complex Figure, Recall – Auditory-Verbal Learning Test, immediate and delayed recall; Lezak et al., 2004) verbal and non-verbal memory, attention and executive functions (Trail Making Test B, A and B-A; Inverted Motor Learning-Clock Drawing Test; Lezak et al., 2004), abstract reasoning thinking (Raven Colored Progressive Matrices; Lezak et al., 2004), frontal functions (Inverted Motor Learning); language (Phonological and Semantic fluency-Token test; Lezak et al., 2004), and apraxia and visuo-constructional abilities (Rey–Osterrieth Complex Figure, Rey figure copy, Clock Drawing Test; Rosen et al., 1980); (v) assessment of depressive symptoms by means of the Center for Epidemiologic Studies Depression Scale (CES-D; Radloff, 1977). All the neuropsychological tests were standardized on Italian population, thus scores were compared to normative values with age, education and gender corrections in an Italian population.

### EEG and MRI

#### EEG Recordings

The EEG activity was recorded, continuously from 19 sites by using electrodes set in an elastic cap (Electro-Cap International, Inc.) and positioned according to the 10–20 international systems (Fp1, Fp2, F7, F3, Fz, F4, F8, T3, C3, Cz, C4, T4, T5, P3, Pz, P4, T6, O1, and O2). The patients were instructed to stay sit with closed eyes and relaxed. In order to keep constant the level of vigilance, an operator controlled on-line the subject and the EEG traces, alerting the subject any time there were signs of behavioral and/or EEG drowsiness. The ground electrode was placed in front of Fz. The left and right mastoids served as reference for all electrodes. The recordings were used off-line to re-reference the scalp recordings to the common average. Re-referencing was done prior to the EEG artifact detection and analysis. Data were recorded with a band-pass filter of 0.3–70 Hz, and digitized at a sampling rate of 250 Hz (BrainAmp, BrainProducts, Germany). Electrodes-skin impedance was set below 5Ω. Horizontal and vertical eye movements were detected by recording the electrooculogram (EOG). The recording lasted 5 min, with subjects with closed eyes. Longer recordings would have reduced the variability of the data, but they would also have increased the possibility of slowing of EEG oscillations due to reduced vigilance and arousal. EEG data were then analyzed and fragmented off-line in consecutive epochs of 2 s, with a frequency resolution of 0.5 Hz. The average number of epochs analyzed was 140, ranging from 130 to 150. The epochs with ocular, muscular and other types of artifacts were discarded by two skilled electroencephalographists (Moretti et al., 2003).

#### Analysis of Individual Frequency Bands

All recordings were obtained in the morning with subjects resting comfortably. Vigilance was continuously monitored in order to avoid drowsiness. A digital FFT-based power spectrum analysis (Welch technique, Hanning windowing function, no phase shift) computed – ranging from 2 to 45 Hz – the power density of EEG rhythms with a 0.5 Hz frequency resolution. Two anchor frequencies were selected according to the literature guidelines (Klimesch, 1997; Moretti et al., 2004), that is, the theta/alpha transition frequency (TF) and the individual alpha frequency (IAF) peak. IAF and TF were computed for each subject in the study. These anchor frequencies were computed on the power spectra averaged across all recording electrodes. This “collapsed spectrum method” allows to identify a robust and reliable IAF, being a normalized scalp spectrum. The TF marks the TF between the theta and alpha bands, and represents an estimate of the frequency at which the theta and alpha spectra intersect. TF was computed as the minimum power in the alpha frequency range, since our EEG recordings were performed at rest. The IAF represents the frequency with the maximum power peak within the extended alpha range (5–14 Hz). Based on TF and IAF, we estimated the frequency band range for each subject, as follows: delta from TF-4 to TF- 2, theta from TF-2 to TF, low alpha band (alpha1 and alpha2) from TF to IAF, and high alpha band (or alpha3) from IAF to IAF + 2. The alpha1 and alpha2 bands were computed for each subject as follows: alpha1 from TF to the middle point of the TF-IAF range, and alpha2 from such middle point to the IAF peak (Fischl et al., 1999; Bazanova and Vernon, 2013). The mean frequency range computed in MCI subjects considered as a whole are: delta 2.9–4.9 Hz; theta 4.9–6.9 Hz; alpha1 6.9–8.9 Hz; alpha2 8.9–10.9 Hz; alpha3 10.9–12.9 Hz. Finally, in the frequency bands determined on an individual basis, we computed the relative power spectra for each subject. The relative power density for each frequency band was computed as the ratio between the absolute power and the mean power spectra from 2 to 45 Hz. The relative band power at each band was defined as the mean of the relative band power for each frequency bin within that band. The alpha3/alpha2 was computed in all subjects and three groups were obtained according to increasing tertiles values of alpha3/alpha2: low (a3/a2 < 1) middle (1 ≤ a3/a2 ≤ 1.16) and high (a3/a2 ≥ 1.17). The three groups of MCI has been demonstrated in previous studies to be different in nature. In particular, the high alpha3/alpha2 EEG power ratio MCI group is at major risk to convert to Alzheimer’s disease (Frisoni, 2012), as well as to have different pattern of hippocampal atrophy (Moretti et al., 2011b) and basal ganglia and thalamus gray matter lesions (Moretti et al., 2009) as compared to the other alpha3/alpha2 power ratio MCI groups. Moreover, this group subdivision has been chosen for reason of homogeneity and comparability with the previous studies. As the aim of our study was to evaluate the relationship only between functional and morphostructural biomarkers in a group of MCI who has major probability to develop AD, we did not consider the clinical subtype of MCI, i.e., amnesic, or non-amnesic, single or multiple domains.

#### MRI Scans

For each subject, a high-resolution sagittal T1 weighted volumetric MR scan was acquired at the Neuroradiology Unit of the ‘Citta` di Brescia’ Hospital, Brescia, by using a 1.0 T Philips Gyroscan scanner, with a gradient echo 3D technique: TR = 20 ms, TE = 5 ms, flip angle = 30, field of view = 220 mm, acquisition matrix 256 ⋅ 256, slice thickness 1.3 mm.

#### Cortical Thickness Estimation Steps

Cortical thickness measurements for 74 MCI patients were made using a fully automated MRI-based analysis technique: FreeSurfer, a set of software tools for the study of cortical and subcortical anatomy. Briefly, in the cortical surface stream, the models of the boundary between white matter and cortical gray matter as well as the pial surface were constructed. Once these surfaces are known, an array of anatomical measures becomes possible, including: cortical thickness, surface area, curvature, and surface normal at each point on the cortex. In addition, a cortical surface-based atlas has been defined based on average folding patterns mapped to a sphere and surfaces from individuals can be aligned with this atlas with a high-dimensional non-linear registration algorithm. The surface-based pipeline consists of several stages previous described in detail (Fischl and Dale, 2000; Gronenschild et al., 2012).

##### Single Subject Analysis

For each subjects the T1-weighted, anatomical 3-D MRI dataset were converted from Dicom format into .mgz format, then intensity variations are corrected and a normalized intensity image is created. The volume is registered with the Talairach atlas through an affine registration. Next, the skull is stripped using a deformable template model (Segonne et al., 2004) and extracerebral voxels are removed. The intensity normalized, skull-stripped image is then operated on by a segmentation procedure based on the geometric structure of the gray–white interface. Voxels are classified as white or gray matter, cutting planes are chosen to separate the hemispheres from each other. A white matter surface is then generated for each hemisphere by tiling the outside of the white matter mass for that hemisphere. This initial surface is then refined to follow the intensity gradients between the white and gray matter. The white surface is then nudged to follow the intensity gradients between the gray matter and CSF, obtaining the pial surface. Cortical thickness measurements were obtained by calculating the distance between those surfaces (white and pial surface) at each of ∼160,000 points per hemisphere across the cortical mantle (Dale et al., 1999).

##### Group Analysis

In order to relate and compare anatomical features across subjects, it is necessary to establish a mapping that specifies a unique correspondence between each location in one brain and the corresponding location in another. Thus, the pial surface of an individual subject is inflated to determine the large-scale folding patterns of the cortex and subsequently transformed into a sphere to minimize metric distortion. The folding patterns of the individual are then aligned with an average folding pattern using a high-resolution surface-based averaging. Thickness measures were mapped to the inflated surface of each participant’s brain reconstruction allowing visualization of data across the entire cortical surface. Finally, cortical thickness was smoothed with a 20-mm full width at half height Gaussian kernel to reduce local variations in the measurements for further analysis.

#### Test–Retest Reproducibility of Cortical Thickness Analysis

Previous studies have investigated the reliability of the cortical thickness measurements: some of these addressed the effect of scanner-specific parameters, including field strength, pulse sequence, scanner upgrade, and vendor. The use of a different pulse sequence had a larger impact, as did different parameters employed in data processing. The within-scanner variability of global cortical thickness measurements reported in previous studies was 0.03–0.07 in average (Rosas et al., 2002; Kuperberg et al., 2003; Pennanen et al., 2005; Han et al., 2006). Scanner upgrade did not increase variability nor introduce bias while measurements across field strength were slightly biased (thicker at 3 T). In the study by Han et al. (2006) the variability was 0.15 and 0.17 mm in average, respectively, for cross-scanner (Siemens/GE) and cross-field strength (1.5 T/3 T) comparisons. The recent study by Gronenschild et al. (2012) also investigated the effects of data processing conditions such as FreeSurfer version, workstation, and Macintosh operating system version. The authors reported significant differences between FreeSurfer version (average: 2.8–3%) and a smaller differences between workstation and operating system version. On the whole, the results suggest that MRI-derived cortical thickness measures are highly reliable, however it is important to keep consistent the MRI parameters and data processing factors within any given structural neuroimaging study (DeCarli et al., 2005; Markesbery et al., 2006; McKhann et al., 2011).

#### Radial Atrophy Mapping for Hippocampal Atrophy Computation

The 3D parametric surface mesh models were created from the manual tracings of hippocampal boundaries (DeCarli et al., 2005). This procedure allows measurements to be made at corresponding surface locations in each subject, which are then compared statistically in 3D (DeCarli et al., 2005). To assess hippocampal morphology, a medial curve was automatically defined as the 3D curve traced out by the centroid of the hippocampal boundary in each image slice. The radial size of each hippocampus at each boundary point was assessed by automatically measuring the radial 3D distance from the surface points to the medial curve defined for individual’s hippocampal surface model.

The analysis of variance ANOVA was performed in order to verify the difference of hippocampal volume among groups.

### EEG and SPECT

#### MCI Patients

Mild cognitive impairment patients were taken from a prospective project on MCI (“Mild Cognitive Impairment in Brescia, MCIBs”), aimed to study the natural history of persons without dementia with apparently primary cognitive deficits, i.e., not due to psychic or physical conditions, the same of MRI-EEG project. The study protocol was approved by the local ethics committee and all participants signed an informed participation consent. Details on inclusion/exclusion criteria and on physical and neurological examinations, performance-based tests of physical function, gait and balance and performed neuropsychological battery have been previously published and are at disposal elsewhere (Caroli et al., 2007; Moretti et al., 2013b) and described above. Among the 56 MCI patients who agreed to undergo MRI and SPECT scan, all consecutive 27 who agreed also to undergo EEG recording were further considered.

#### Normal Controls

We enrolled all 17 healthy subjects from a previous study on cerebral perfusion correlates of conversion to AD with both an MRI and a SPECT scan available (Caroli et al., 2007; Moretti et al., 2013b). Briefly, subjects were consecutive normal volunteers picked among those undergoing brain MRI scan at the Neuroradiology Unit of the “Città di Brescia” Hospital in Brescia from October 2004 to June 2006 for reasons unrelated to cognition, or were healthy volunteers aged 65 years or older, among MCI patients’ spouses, friends of them, and researchers’ acquaintances. All scans of enrolled subjects were normal on visual assessment by a neuroradiologist. Subjects underwent multidimensional assessment including clinical, neurological, and neuropsychological evaluations, and drawing of a blood sample (not used for the purposes of the present study). Data coming from normal controls were used only to compute *W* scores in each selected perfusion Region of Interest (ROI).

### SPECT Scan

Both patients and normal controls underwent SPECT scan in the nuclear medicine department of the Ospedali Riuniti in Bergamo. Each patient received an intravenous injection of 925 MBq of technetium- 99 m ethylcysteinate dimer (99mTc-ECD) in resting conditions, lying supine with eyes closed in a quiet, dimly lit room. Forty to sixty minutes after injection, brain SPECT was performed using a dual-head rotating gamma camera (GE Elscint Helix) equipped with low energy-high resolution, parallel hole collimators. A 128 × 128 pixel matrix, zoom = 1.5, was used for image acquisition with 120 views over a 360^°^ orbit (in 3^°^ steps) with a pixel size and slice thickness of 2.94 mm. Butterworth filtered-back projection (order = 7, cut-off = 0.45 cycles/cm) was used for image reconstruction, and attenuation correction was performed using Chang’s method (attenuation coefficient = 0.11 cm-1). Images were exported in DICOM format.

Flow chart summarizing the whole MRI-SPECT processing protocol.

#### SPECT Processing Protocol

**FIGURE 1:**
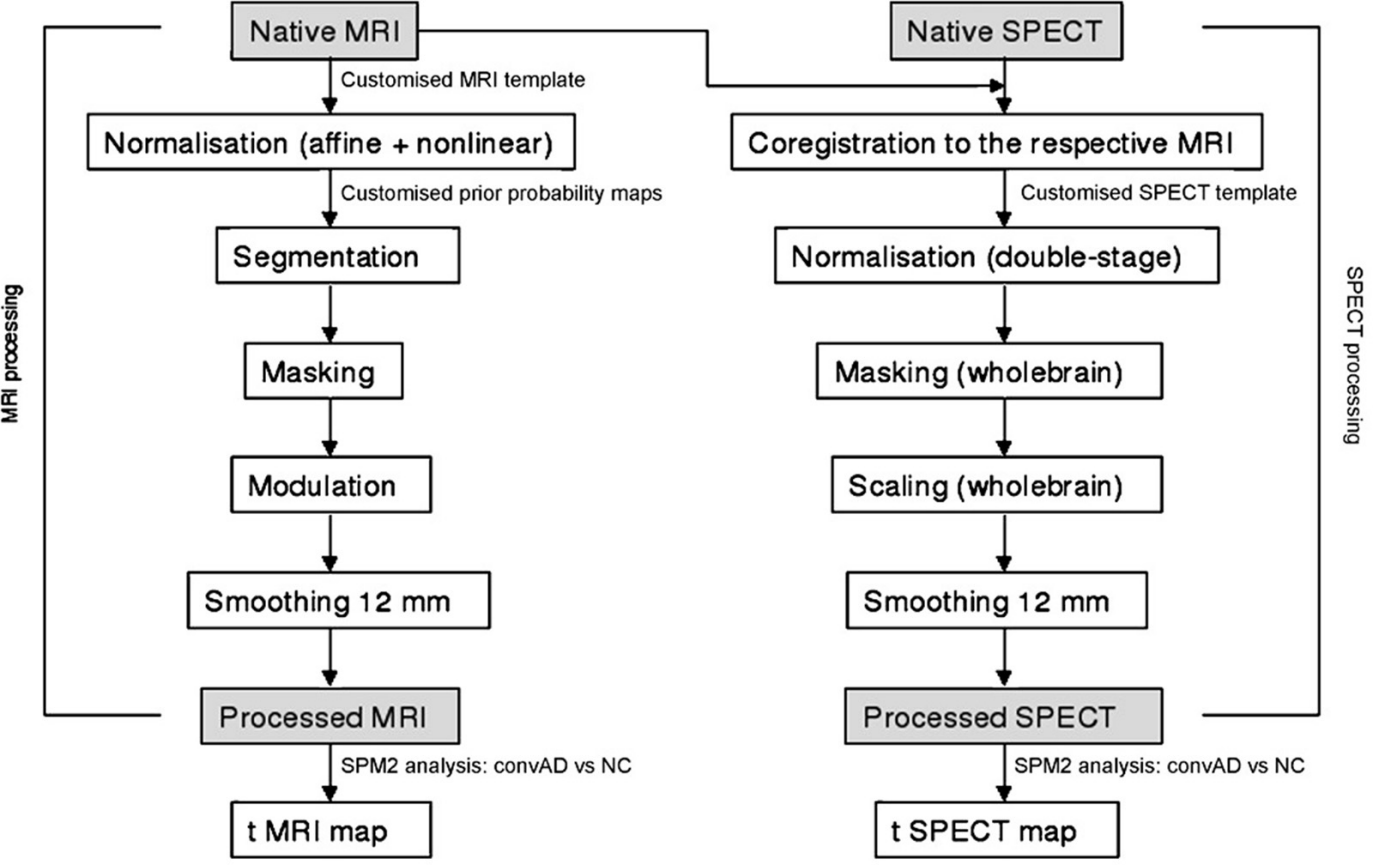
**Flow chart summarizing the whole MRI-SPECT processing protocol**.

To achieve a precise normalization, we generated a study-specific SPECT template using both SPECT and MRI scans of all patients and normal controls under study, following a procedure described in detail elsewhere (Caroli et al., 2007) and schematically represented in **Figure 1**. Briefly, we created a customized high-definition MRI template, we converted SPECT scans to Analyze format using MRIcro (Rorden and Brett, 2000), and we coregistered them to their respective MRI scans with SPM2 (SPM, Statistical Parametric Mapping, version 2,2002. London: Functional Imaging Laboratory. Available at: http://www.fil.ion.ucl.ac.uk/spm/software/spm2). We normalized each MRI to the customized MRI template through a non-linear transformation (cut-off 25 mm), and we applied the normalization parameters to the coregistered SPECT. We obtained the customized SPECT template as the mean of all the latter normalized SPECT images. The creation of a study-specific template allows for better normalization, since low uptake in ventricular structures and cortical hypoperfusion effects are frequently present in elderly patients. For each coregistered SPECT scan, we set the origin to the anterior commissure, using the respective MRI image as a reference, and we processed all scans with SPM2 according to an optimized processing protocol described in detail elsewhere (Caroli et al., 2007). Brain perfusion correlates of medial temporal lobe atrophy and white matter hyperintensities in MCI were obtained as follows: (I) we smoothed each scan with a 10 mm full width at half maximum (FWHM) Gaussian, and spatially normalized it with an affine deformation to the customized SPECT template; we applied the same deformation to the unsmoothed images; (II) we masked the unsmoothed normalized images from I to remove scalp activity using SPM2’s “brainmask.” We smoothed with a 10 mm FWHM Gaussian, and warped them to the customized template with a non-linear transformation (cut-off 25 mm); we applied the same transformation to the unsmoothed masked images; (III) we smoothed the normalized unsmoothed images from II with a 12 mm FWHM Gaussian. The following ROI were chosen for perfusion analyses in each hemisphere from the Pick atlas by a sub-routine implemented on SPM2: frontal, parietal and temporal lobes, the thalamus and the hippocampal-amygdalar complex (Maldjian et al., 2003). The choice of these regions was based on previous SPECT and PET studies in subjects with MCI (Staffen et al., 2009; Alegret et al., 2012; Yoon et al., 2012).

The whole cerebellum was chosen for normalization of ROI counts. Since perfusion values in selected ROIs did not account for age, pertinent age corrected perfusion values (hereafter called *W* scores), were computed in each selected ROI, following a previously published procedure (Alegret et al., 2012).

### Statistical Analysis for MRI and EEG

Differences between groups in sociodemographic and neuropsychological features were analyzed using SPSS version 13.0 (SPSS, Chicago, IL, USA) performing an analysis of variance (ANOVA) for continuous variables and paired χ^2^ test for dichotomous variables. For continuous variables, post-hoc pairwise comparisons among groups were performed with the Games-Howell or Bonferroni tests depending on homogeneity of variance tested with Levene’s test.

Concerning the neuroimaging analysis, the Qdec interface in Freesurfer software was used: a vertex-by-vertex analysis was carried out performing a general linear model to analyze whether any difference in mean cortical thickness existed between groups (low a3/a2 < 1 μV^2^;) middle (1 ≤ a3/a2 ≤ 1.16 μV^2^ and high (a3/a2 ≥ 1.17 μV^2^). The following comparisons were carried out: High vs. Low, High vs. Middle, and Middle vs. Low. Age, sex, education, global cognitive level (MMSE score) The value of cortical thickness estimation in middle and low was averaged and compared to the high alpha3/alpha2 power ratio. When a statistical threshold at *p* ≤0.05 corrected was applied, there were no significant results. So we choose to apply an uncorrected but more restrictive significance threshold than 0.05 (*p*≤ 0.001) and we considered as significant only the clusters which also were wide equal or major to 30 mm^2.^ Finally a surface map was generated to display the results on an average brain. For illustrative purpose significance was set to a *p*-value of ≤ 0.01 uncorrected for multiple comparisons.

### Statistical Analysis for SPECT AND EEG

All statistical analyses were performed using SPSS software version 13.0. We investigated significance of the difference between the two groups (MCI at low and at high risk to develop AD) in socio-demographic, clinical and cognitive features using χ2 test for categorical variables (sex, and ApoE carriers) and Student’s independent *t* test for continuous variables (volumetric, perfusion features and EEG frequencies). In all cases we set the significant threshold at *p* < 0.05. Since native SPECT scans were coregistered to their respective MRI images, and the study-specific SPECT template was coregistered to the high-definition MRI template, all the normalized SPECT and MRI images used for the statistical analysis were coregistered to the SPM standard anatomical space. Moreover, Pearson’s r correlations were assessed between the selected perfusion ROIs (in terms of age corrected *W* scores) and the acquired EEG frequencies in both groups. Moreover, a correlation analysis was computed between theta and alpha brain rhythms.

## Results

### MRI-EEG

**Table 1** shows the sociodemographic and neuropsychological characteristics of MCI subgroups defined by the tertile values of alpha3/alpha2. The ANOVA analysis showed that there was not statistically significant differences between groups which resulted well paired for age, sex, education, and global cognitive level. Anyway, age, sex, education, global cognitive level (MMSE score) alpha3/alpha2 ratio levels were significant at Games-Howell *post hoc* comparisons (*p* = 0.000).

#### Pattern of Cortical Thickness between Groups

**FIGURE 2:**
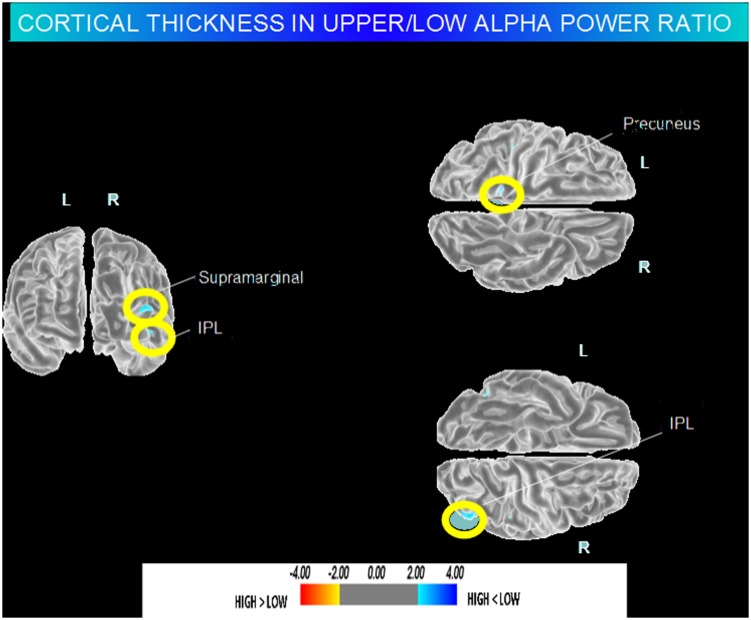
**Brain regions with significant regional cortical thickness differences in MCI with high a3/a2 ratio compared to MCI with low averaged with middle a3/a2 ratio (*p* < 0.01 uncorrected)**. The color-coding for *p* values is on a logarithmic scale. Warmer color represents cortical thinning, cooler color represents cortical thickening. Results are presented on the pial cortical surface of brain: dark gray regions represent sulci and light gray regions represent gyri.

High vs. Middle and Low averaged thickness (named low): when compared to subjects with low a3/a2 ratios, patients with high a3/a2 ratio show thinning in the right Supramarginal and IPL and in the left Precuneus cortex, (**Figure 2**; **Table 2**).

**Table 2:** Brain regions with significant regional cortical thickness differences in MCI with high a3/a2 ratio compared to MCI with low averaged with middle a3/a2 ratio.

High a3/a2<averaged middle and low a3/a2								
Cluster size (mm^2^)	Region	Side	Stereotaxc coordinate			*P*	Thickness (mm^2^)	
			***x***	***y***	***z***		**High**	**Low+middle**
55	Precuieus	L	-14	-48	58	0.00001	1.35 ± 0.14	2.57 ± 0.24
76	Supra marginal	R	49	-29	27	00.01.00	1.53 ± 0.18	2.67 ± 0.56
93	Inferior parietal	R	46	-75	10	0.001	1.54 ± 0.22	3.07 ± 0.35

*Cluster size represents the extension of contiguous significant voxels in the cluster obtained at *p* < 0.01 uncorrected (cluster size > 30 mm^2^). Stereotaxic coordinates reveal the position of the most significant voxel of the cluster, and side denotes its localization on the left (L) or right (R) brain hemisphere. Thickness denotes the average cortical thickness and SD values within the cluster in high and low + middle a3/a2 groups. P denotes the significance level of the differences in thickness between groups*.

### SPECT-EEG

Twenty seven MCI patients were enrolled for the present study and they were classified as at high risk (when the a3/a2 EEG rhythm median was above 1.17) or at low risk (when the a3/a2 EEG rhythm median was under 1.17) to develop AD. The two groups (AD high risk, *N* = 13, AD low risk, *N* = 14) were similar for age (*p* = 0.56), education in years (*p* = 0.87), gender (*p* = 0.17), ApoE genotype (*p* = 0.15), MMSE scores (*p* = 0.31) and white matter lesions load (*p* = 0.88; **Table 3**). **Figure 1** shows the visual rating scale of the SPECT scans representative of normal control, MCI with low and MCI with high risk to convert in AD, respectively. ANOVA results show that the selected cut-off was effective in detecting two different groups: patients with high risk to develop AD show significantly higher alpha3/alpha2 power ratio than patients with low risk (*p* = 0.0001). Moreover, a control analysis was performed on the single frequencies. The results show that the increase of alpha3/alpha2 frequency power ratio was due to both increase of alpha3 (*p* = 0.001) and decrease of alpha2 (*p* = 0.0001) and not to the modification of a single frequency. This control analysis was performed because the change of only one frequency could be due to the chance. But it was not the case.

**Table 3:** Demographic and cognitive characteristics in the whole sample, disaggregated for increased levels of alpha3/alpha2 Numbers denote mean ± SD, number, and [range].

	At low-risk MCI	At high-risk MCI	*P* value
N	14	13	
Age (years) [range]	69.1 ± 7.6 [57÷83]	70.6 ± 5.5 [62÷78]	0.555
Gender (females)	6 (43%)	9 (69%)	0.168
Education (years) [range]	8.2 ± 4.3 [4-18]	7.9 ± 4.5 [3÷18]	0.865
MMSE score [range]	27.9 ± 1.6 [25÷30]	27.2 ± 1.9 [24÷29]	0.309
3 Left hippocampal volume (mm^3^) [Range]	2,606 ± 353 [1,923÷3,017]	2,073 ± 412 [1,234÷2,641]	0.001
3 Right hippocampal volume (mm^3^) [range]	2,581 ± 473 [1,549÷3,150]	2,296 ± 501 [1,589÷3,086]	0.141
Wahlund total score [Range]	3.58 ± 3.29 [0.0÷10.0]	3.78 ± 2.63 [0.0÷7.0]	0.886

*p denotes significance on ANOVA*.

**FIGURE 3:**
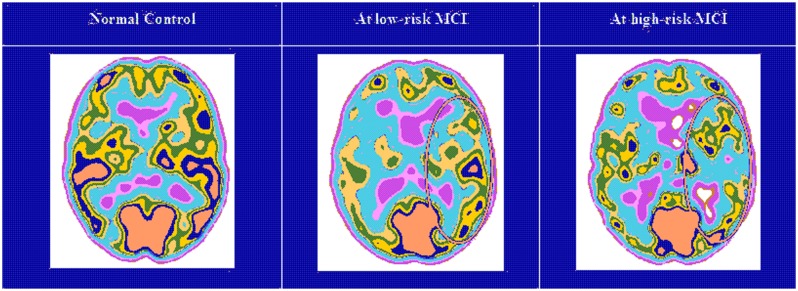
**Single-photon emission computed tomography (SPECT) visual rating**. The output shows a SPECT visual inspection of glucose uptake metabolism: the white square denotes an area of mild-to-moderate (purple to blue) temporparietal hypometabolism in one of the 14 at low risk and in one of the 13 at high risk MCI patient respect to one of the 17 enrolled controls.

Although the mean perfusion in all the selected ROIs was similar between groups (all *p* > 0.38), in the group with high alpha3/alpha2 frequency ratio there is a constant trend to a lower perfusion (see **Figure 3**). Moreover, left hippocampal volumes were lower for AD-high risk patients respect to low risk ones (*p* < 0.001; **Table 3**). Data coming from normal controls were used only to compute *W* scores in each selected perfusion ROI. In patients at low risk to develop AD, significant Pearson’s R negative correlation was found between perfusion in the hippocampal complex ROI and theta rhythm (*r* = -0.544, *p* = 0.044).

In patients at high risk to develop AD otherwise, more and dissimilar correlations were found: a positive correlation, inverted respect to patients at low risk, between the perfusion in the hippocampal complex ROI and theta rhythm (*r* = 0.729, *p* = 0.005). No other significant correlations were found in both groups between perfusion ROIs and other EEG rhythms or hippocampal volumes. Moreover, no significant correlations were found between hippocampal complex ROI and theta rhythm pooling low and high risk patients together (*r* = 0.086, *p* = 0.671). The correlation analysis between theta and alpha rhythm showed a positive correlation between the ghigher alpha3/alpha2 power ratio and the theta brain rhythms (*r* = 0.67, *p* < 0.03).

## Discussion

### Association between EEG Markers and MRI Changes

In the present study the relationship between an EEG marker (the alpha3/alpha2 power ratio) and the cortical thickness in subjects with MCI was investigated. The alpha3/alpha2 power ratio has been chosen because in previous works it has been demonstrated that MCI subjects with higher alpha3/alpha2 ratio are at major risk to develop AD (Moretti et al., 2009, 2011b, 2013b; Frisoni, 2012). Our results show that the MCI group with higher alpha3/alpha2 ratio has a greater global cortical atrophy than the other subgroups, thus confirming a large body of literature (Frisoni et al., 2007; Frisoni, 2012). Furthermore, the greater atrophy is significant in two specific brain areas: precuneus and supramarginal gyrus (a brain area belonging to the inferior parietal lobule), both in left and right hemisphere. These results was largely expected considering previous studies. Indeed, structural and functional abnormalities of the precuneus were observed in MCI (Ryu et al., 2010) as well a in Azheimer’s disease (Sperling et al., 2010) so that the atrophy of precuneus has been considered as a pathognomonic marker of early AD. Recent studies suggest that the pathophysiological process of AD exerts specific deleterious effects on distributed memory circuits, even prior to clinical manifestations of significant memory impairment. Specific regions, namely the precuneus and posterior cingulate, together with the medial temporal lobe, are selectively vulnerable to early amyloid deposition in AD pathology (Sperling et al., 2010; De Haan et al., 2012). Recent studies have demonstrated that during the successful encoding of new items there is a desynchronization in the temporo-parietal memory-related networks whereas a synchronization prevent a successful semantic encoding (Ryu et al., 2010; Pievani et al., 2011). The deleterious role of synchronization has been recently demonstrated by an interesting study facing the intriguing relationship between functional and structural degeneration in AD (Sperling et al., 2010). The authors detected some hub regions (heteromodal associative regions) selectively vulnerable in AD pathology, due to the damage of inhibitory interneurons providing a loss of inhibition at cellular level. According to the authors, the disinhibition provokes an increasing amount of neural activity at network level, giving as a final result an hypersynchronization of brain areas. Of note, this overactivity is excitotoxic and determines cellular apoptosis and brain atrophy. Also, Palop and Mucke (2010) emphasize the role of inhibitory interneuron dysfunction, leading to hypersynchronization (Jones et al., 2011; Brier et al., 2012; Chatwal and Sperling, 2012). Our results are in line with these previous influential studies. A possible integrative view of all the results could be as follows: (1) the higher neuronal activity in the hub regions starts from a disfunction of cellular inhibition; (2) the consequent disinhibition drives neural network to an oversynchronization; (3) this oversynchronization is peculiar of the hub regions with higher amyloid burden; (4) these overactivated regions are prone to degeneration and atrophy; (5) a possible neurophysiologic sign of this oversynchronization is the increase of the alpha3/alpha2 power ratio we have found in typical hub regions (Stam et al., 2003; Rossini et al., 2008; Bhattacharya et al., 2011; Wu et al., 2011). It is of great interest that there is an overlapping between the brain regions associated with increase of EEG alpha3/alpha2 power ratio (hypersynchronization of upper alpha) in our study and the regions associated with higher amyloid burden related to memory processes (Palop and Mucke, 2010; Chatwal and Sperling, 2012). Moreover, in the present study, there is a very interesting result. The atrophy of precuneus is coupled with the atrophy in supramarginal gyrus and, at lesser extent, with inferior parietal, insula and superior temporal gyrus. This atrophy pattern is clearly expressed in the group of MCI subjects with higher alpha3/alpha2 power ratio. This finding fits well with the results of a recent study (Wonderlick et al., 2009), investigating the functional connectivity of human precuneus by resting state fMRI. The authors found that there is a preferential pathway of connectivity of the dorsal precuneus with supramarginal gyrus, parietal cortex, superior temporal gyrus and insula. As a consequence, the atrophy we detected in the MCI group with higher alpha3/alpha2 ratio power could be hypothesized as the loss of GM in an entire anatomo-functional network more than atrophy of isolated brain areas. Of note, it is widely accepted that AD is the result of a cortical network impairment more than the atrophy of single cortical areas (Zhang and Li, 2012). In subjects with low or middle alpha3/alpha2 power ratio the cognitive impairment is possibly due to cerebro-vascular impairment or non-AD degenerative process. Although rigid selection criteria were adopted to include in the study only patients with primary cognitive deficits, in the clinical practice is not infrequent to have MCI subjects not due to AD.

### Association between EEG Markers and SPECT Changes

The EEG alpha3/alpha2 frequency ratio in previous studies has proved useful in identifying a group at greater risk of converting in AD (Stam et al., 2005). This group has the higher alpha3/alpha2 EEG frequency power ratio, at an orientative cut-off of about 1.17. The choice of a cut-off allows the individuation of a particular population inside the group of patients with MCI. It is a very important issue of the study and makes it different from other works, usually distinguishing the MCI subjects on clinical, structural or functional aspects but not on a neurophysiological marker. The particular group individuated by the higher alpha3/alpha2 power ratio is at major risk to develop AD. The possibility to detect this risk not only in a group but also in the single patient through a cut-off is also an original contribution of this study. To be validated this EEG marker needs correlation study with morphostructural or functional milestones peculiar of AD, like as rCBF. These present results confirm that the relationship alpha3/alpha2 identifies two distinct groups: the higher ratio characterizes a group with a smaller hippocampal volume and a constant trend of lower cerebral perfusion, even if it does not reach significance. These results confirm previous studies which have shown that patients with high risk of developing AD have left hippocampal atrophy and reduced SPECT perfusion (Frisoni, 2012). Actually, amyloid plaques deposition, NFT formation, neuronal loss, decrease in dendritic extent, and synaptic depletion are thought to disturb the communication among various cortical areas, resulting in anatomic isolation and decreased perfusion of many cortical zones (Golde, 2003). The lack of a significant difference is an obvious limitation of the work. One possible explanation is the relatively small sample size of the two groups. Given that the trend is constant, a larger sample in both groups could exploit a significant statistical difference. On the other side, it is possible that when considering two groups of patients, both with a MCI, the rCBF is not so sensible to evidence little difference. On the contrary, previous studies have demonstrated that metabolic, but not perfusional, patterns were related to severity of cognitive impairment and were more sensible in detecting prodromal MCI due to AD (Mielke et al., 1994). Further studies, with larger sample size, are mandatory to confirm these results. The present study shows a correlation between cerebral perfusion and theta rhythm. Anyway, the correlation emerges only when considering the different groups individuated on the alpha3/alpha2 frequency power ratio. This is confirmed by the finding that when the groups are merged, no correlation could be found. This is the main aspect of the study and the peculiar novelty of the results. The patients at lower risk to develop AD, who have a constant trend toward a higher brain regional blood perfusion, maintains low levels of hippocampal theta power while in patients at higher risk, with a basically lower cerebral blood perfusion, theta rhythm tends to be higher. This latter finding is also confirmed by the increased ratio of theta/gamma frequency power ratio in the temporal region, adjacent to the hippocampus. A lot of previous studies have shown an increase of theta rhythm in patients with mild AD (Rodriguez et al., 2011), so that the increase of theta power is a robust features of AD. Theta rhythms are usually not appreciated in normal awakening EEG. However, a theta power increase is observed over the frontal and temporal areas during learning and memory tasks. The theta rhythms that are recorded during these tasks are thought to be produced by the activation of septal-hippocampal system. On the other hand, it should be taken in mind that EEG measures electrical field variations, and a number of clinical conditions can disturb the normal electrical field of the brain. For instance, electrolyte changes may alter the appearance and time variation of the brain-generated electrical fields, and medications can slow the posterior dominant rhythm. Moreover, in assessing the frequency of the theta rhythm, cerebrovascular lesions should be considered as a possible cause of increase. By means of observations in patients with ischemic lesions, it has been suggested that delays in corticocortical fiber propagation may play a global role in determining human EEG frequencies, increasing the amount of theta activity (Thatcher et al., 1998). Increased T2 relaxation times in cortical gray matter and white matter were correlated with a shift in relative EEG power to lower frequencies in the theta range (4–7 Hz) and reduced cognitive performance (Rodriguez et al., 2011). Anyway, none of our patients suffered from acute ischemic lesions and there was no difference in the cerebrovascular load between the two groups. Moreover, the EEG frequency details of patients with chronic cerebrovascular load has been recently investigated (Moretti et al., 2004) and they are not compatible with an high alpha3/alpha2 frequency ratio increase. So, we are confident the our results are of neurodegenerative origin. On the whole, it emerges a picture in which it is not the simple cerebral blood perfusion rate nor a single brain rhythm that reflect the complexity of functional alteration in AD. A previous work already found that none of the regions of interest of the SPECT scans were significantly correlated with clinical severity (Müller et al., 1997; Wenderoth et al., 2005).

### Theta and Alpha Frequency Interplay in MCI Due to AD

Klimesch et al. (1996) and Klimesch (1997, 1999) have convincingly demonstrated that that the upper alpha band (10–13 Hz) specifically reflects encoding memory processes. Recent EEG and magnetoencephalography (MEG) studies have confirmed that a correct functioning of memory, both in encoding and in retrieval, requires the high alpha rhythm desynchronization (or power decrease; Kilner et al., 2005; Wyart and Tallon-Baudry, 2008; Spitzer et al., 2009; Staudigl et al., 2010; Moretti et al., 2012b). From a neurophysiological point of view the synchronization (or power increase) of EEG alpha power has been associated with the inhibition timing hypothesis (Moretti et al., 2012b) and with poor information transmission, according to he entropy’s theory (Hanslmayr et al., 2010; Moretti et al., 2013b). The increases in alpha amplitudes reflect inhibition of cortical brain regions (Hanslmayr et al., 2012; Moretti et al., 2012a, 2013a). Similarly, the entropy’s theory stated that synchronization is disadvantageous for storing information, as it reduces the flow of information (Moretti et al., 2013b). Entropy is a measure of the richness of information encoded in a sequence of events. Applying this concept to the neural networks, it has been demonstrated (Wonderlick et al., 2009) that the degree of information that is encoded in neural assemblies increases as a function of desynchronization and decreases as a function of synchronized firing patterns (Jensen and Mazaheri, 2010; Norman, 2010). This hypothesis has been confirmed in clinical studies in patients with memory deficits (Schneidman et al., 2011). as well as during states where there is little cognitive processing (e.g., epileptic seizures or slow wave sleep; Goard and Dan, 2009; Wonderlick et al., 2009; Kurimoto et al., 2012) As regards cognitive impairment due to AD, the typical synaptic loss could prevent the physiological flexibility of brain neural assemblies, impeding the desynchronizing downstream modulation of the brain activity. As a consequence, it could be hypothesized that the disruption of cortical network due to degenerative disease, inducing cortical atrophy, could determine an oversynchronization of the brain oscillatory activity. The synchronization state of the high alpha power could prevent the creation of a semantic sensory code and, consequently, of the episodic memory trace (Barlow, 1961; Bialek et al., 1991; Bazanova and Aftanas, 2008; Chalk et al., 2010). In previous seminal studies, high alpha frequency has been specifically related to semantic memory processes (Craik, 2002; Moretti et al., 2003; Hanslmayr et al., 2009). Of note, in subjects with early cognitive decline, the impairment of the semantic features of memory has been recently accepted as a hallmark for the early AD diagnosis. (Dubois et al., 2007; Albert et al., 2011). Indeed, according to the new diagnostic criteria of AD, the measurement of sensitivity to semantic cueing can successfully differentiate patients with AD from healthy controls, even when patients are equated to controls on MMSE scores or when disease severity is very mild. Our results are generally in line with this hypothesis, suggesting that increase in power of high alpha brain oscillations reflects a block of information processes. However, the present study goes one step further, linking the increase of high alpha synchronization to the atrophy of a specific brain network, correlated with impairment in memory performances. Hippocampus has a cholinergic innervation originating from basal forebrain, the medial septum, and the vertical limb of the diagonal band of Broca. Populations of GABAergic and glutamatergic neurons have also been described in several basal forebrain structures. The synchronized depolarization of hippocampal neurons produces field potentials that have a main frequency of 3–12 Hz and are usually known as hippocampal theta rhythm (Bland and Colom, 1993; Craik, 2002). A cholinergic–glutamatergic hypothesis of AD, in which most symptoms may be explained by cholinergic–glutamatergic deficits, has been advanced. Neuronal injury/loss may include an excitotoxic component that possibly contributes to the early cholinergic deficit. This excitotoxic component may occur, at least in part, at the septal level where somas of cholinergic neurons are found. This insult may modify septal networks and contribute to the abnormal information processing observed in AD brain, including its hyperexcitability states. According to this theory, the increased theta production in AD would derive from hyperexcitability of the septal-hippocampal system (Bland and Colom, 1993; Colom, 2006; Moretti et al., 2007, 2008). Of note, such pattern of decreased cerebral blood flow activity and increased excitability was found even prior to the onset of cognitive impairment and cortical atrophy (Moretti et al., 2012a).

A recent study, confirms the major role of the interplay of theta and alpha frequency in the cognitive impairment evaluating the global field synchronization and power spectral analysis (Abuhassan et al., 2014).

This study have investigated the interplay between various synaptic degeneration and compensation mechanisms, and abnormal cortical oscillations based on a large-scale network model consisting of 100,000 neurons exhibiting several cortical firing patterns, 8.5 million synapses, short-term plasticity, axonal delays and receptor kinetics.

The structure of the model is inspired by the anatomy of the cerebral cortex. The results of the modeling study suggest that cortical oscillations respond differently to compensation mechanisms. In particular, the local compensation preserves the baseline activity of theta and alpha oscillations. Deactivating local compensation mechanisms will result in rapid decline (cognitive deficit) of the network dynamics at theta and alpha bands. Therefore, methods which can enhance local compensation could play a major role in the stimulation of neural processes and cognitive functions that are associated with these frequency bands. As compensating for synaptic loss is speculated to differ from one cortical area to another, the study suggests that activating an inappropriate compensation mechanism in a particular area may fail to recover the network dynamics and/or may induce secondary pathological changes in the network. This speculation is supported by the observation that local compensation fails at recovering/maintaining the baseline delta and beta oscillations whilst theta and alpha oscillations are least preserved with global compensation.

### Clinical Implications

The associations between neurophysiological, functional and morphostructural biomarkers may open new perspectives in terms of early diagnosis of Alzheimer’s disease. In addition, the correlation of these biomarkers with peculiar cognitive performance can be a valuable prognostic tool and a mean to identify a particular group of subjects with MCI who may participate in clinical trials in which new therapies are tested. This would allow a more accurate diagnosis, better planning for the future by the patient and his family, and optimization of health care spending. Of course, the next step is to move away from population studies to studies on single subject.

### Study Limitations

There are some limitations due to the obvious explorative nature of the present study: (1) further studies are needed to confirm our result on larger samples and applying an appropriate multiple comparison correction; (2) the pattern of cortical thickness should be investigated on the remaining EEG frequency measures; (3) finally the retrospective nature of the study prevented a direct assessment of whether subjects with increase of a3/a2 EEG power ratio will convert to Alzheimer’s or other neurodegenerative disease; (4) the conservative *p* < 0.001 used here is not necessarily sufficient given the number of comparisons. Anyway, given the explorative nature of the study it is plausible a permissive approach in order to avoid to reject possibly interesting results.

It remains clear that further studies with less permissive statistical approach are mandatory to confirm results.

## Conclusion

The present results show that that synchronization (or increase in power) of high alpha is associated with greater cortical atrophy. The greater cortical atrophy is present both the whole brain volume and in a peculiar memory-related network, including precuneus and temporo-parietal cortices. The combination of EEG alpha3/alpha2 ratio and cortical thickness measure could be useful for identifying individuals at risk for progression to AD dementia and may be of value in clinical context.

## Disclosure Statement

I declare that appropriate approval and procedures were used concerning human subjects.
